# Direct vs. Expressed Breast Milk Feeding: Relation to Duration of Breastfeeding

**DOI:** 10.3390/nu9060547

**Published:** 2017-05-27

**Authors:** Wei Wei Pang, Jonathan Y. Bernard, Geetha Thavamani, Yiong Huak Chan, Doris Fok, Shu-E Soh, Mei Chien Chua, Sok Bee Lim, Lynette P. Shek, Fabian Yap, Kok Hian Tan, Peter D. Gluckman, Keith M. Godfrey, Rob M. van Dam, Michael S. Kramer, Yap-Seng Chong

**Affiliations:** 1 Department of Obstetrics and Gynaecology, Yong Loo Lin School of Medicine, National University of Singapore and National University Health System, Singapore 119228, Singapore; obggt@nus.edu.sg (G.T.); obglnld@nus.edu.sg (D.F.); michael.kramer@mcgill.ca (M.S.K.); 2 Singapore Institute for Clinical Sciences (SICS), Agency for Science, Technology and Research (A*STAR), Singapore 117609, Singapore; Jonathan_Bernard@sics.a-star.edu.sg (J.Y.B.); Soh_Shu_E@sics.a-star.edu.sg (S.-E.S.); paeshekl@nus.edu.sg (L.P.S.); pd.gluckman@auckland.ac.nz (P.D.G.); 3 Department of Biostatistics, Yong Loo Lin School of Medicine, National University of Singapore and National University Health System, Singapore 119228, Singapore; medcyh@nus.edu.sg; 4 Department of Neonatology, KK Women’s and Children’s Hospital, Singapore 229899, Singapore; chua.mei.chien@singhealth.com.sg; 5 Department of Child Development, KK Women’s & Children’s Hospital, Singapore 229899, Singapore; lim.sok.bee@singhealth.com.sg; 6 Department of Paediatrics, Yong Loo Lin School of Medicine, National University of Singapore and National University Health System, Singapore 119228, Singapore; 7 Khoo Teck Puat-National University Children’s Medical Institute, National University Health System, Singapore 119228, Singapore; 8 Department of Paediatric Endocrinology, KK Women's and Children's Hospital, Singapore 229899, Singapore; fabian.yap.k.p@singhealth.com.sg; 9 Department of Maternal Fetal Medicine, KK Women’s and Children’s Hospital, Singapore 229899, Singapore; tan.kok.hian@singhealth.com.sg; 10 Duke-NUS Medical School, Singapore 169857, Singapore; 11 Liggins Institute, University of Auckland, Auckland 1142, New Zealand; 12 Medical Research Council Lifecourse Epidemiology Unit, Southampton SO16 6YD, UK; kmg@mrc.soton.ac.uk; 13 NIHR Southampton Biomedical Research Centre, University of Southampton and University Hospital Southampton NHS Foundation Trust, Southampton SO16 6YD, UK; 14 Saw Swee Hock School of Public Health, National University of Singapore and National University Health System, Singapore 117549, Singapore; rob.van.dam@nus.edu.sg; 15 Department of Medicine, Yong Loo Lin School of Medicine, National University of Singapore and National University Health System, Singapore 119228, Singapore; 16 Department of Epidemiology, Biostatistics and Occupational Health, Faculty of Medicine, McGill University Faculty of Medicine, Montreal, QC H3A 1A2, Canada

**Keywords:** direct breastfeeding, breast milk expression, duration of breastfeeding, Asian

## Abstract

Background: Studies examining direct vs. expressed breast milk feeding are scarce. We explored the predictors of mode of breastfeeding and its association with breastfeeding duration in a multi-ethnic Asian population. Methods: We included 541 breastfeeding mother—infant pairs from the Growing Up in Singapore Toward healthy Outcomes cohort. Mode of breastfeeding (feeding directly at the breast, expressed breast milk (EBM) feeding only, or mixed feeding (a combination of the former 2 modes)) was ascertained at three months postpartum. Ordinal logistic regression analyses identified predictors of breast milk expression. Cox regression models examined the association between mode of breastfeeding and duration of any and of full breastfeeding. Results: Maternal factors independently associated with a greater likelihood of breast milk expression instead of direct breastfeeding were Chinese (vs. Indian) ethnicity, (adjusted odds ratio, 95% CI; 3.41, 1.97–5.91), tertiary education (vs. secondary education or lower) (2.22, 1.22–4.04), primiparity (1.54, 1.04–2.26) and employment during pregnancy (2.53, 1.60–4.02). Relative to those who fed their infants directly at the breast, mothers who fed their infants EBM only had a higher likelihood of early weaning among all mothers who were breastfeeding (adjusted hazard ratio, 95% CI; 2.20, 1.61–3.02), and among those who were fully breastfeeding (2.39, 1.05–5.41). Mothers who practiced mixed feeding, however, were not at higher risk of earlier termination of any or of full breastfeeding. Conclusions: Mothers who fed their infants EBM exclusively, but not those who practiced mixed feeding, were at a higher risk of terminating breastfeeding earlier than those who fed their infants directly at the breast. More education and support are required for women who feed their infants EBM only.

## 1. Introduction

WHO recommends exclusive breastfeeding for the first six months of life, with continued breastfeeding for two years and beyond [1,2]. Breastfeeding in this context is defined as the provision of breast milk, regardless of how breast milk is delivered to the child. As such, most studies on breastfeeding to date have not considered the method in which the infant is fed breast milk, i.e., either directly at the breast or fed expressed (manually or by pump) breast milk via a bottle/spoon/cup.

In many parts of the world, feeding a child expressed breast milk is common. A national survey in the U.S. indicated that 85% of breastfeeding mothers fed their infants some expressed human milk between 1.5 and 4.5 months postpartum [3]. In another study conducted in Shanghai, 64% of breastfeeding mothers expressed breast milk at six weeks postpartum [4]. Various reasons appear to explain the ubiquity of breast milk expression, including the need for women to return to work postpartum [5,6], problems with direct breastfeeding, and enabling a partner or other caretaker to feed the infant [7]. Recent technological advancements in breast pumps have also facilitated breast milk expression [8,9]. 

Despite the rise in expressed breast milk feeding [10,11,12], information is sparse on its impact on breastfeeding duration and on infant health outcomes. Few studies have examined associations between expressed breast milk feeding and breastfeeding duration, and the results have been contradictory [7]. For example, one study from Australia reported that expression of breast milk in the early postpartum period was associated with shorter breastfeeding duration [13]. In another study, however, the expression of breast milk was associated with a longer duration of breastfeeding [14]. Even less is known about the impact of partial vs. exclusive expressed breast milk feeding on breastfeeding duration.

We have previously reported that breast milk expression is common in the multi-ethnic Asian Singapore population [15]. While the majority of mothers who fed their infants expressed breast milk also fed their infants directly at the breast, we found that a substantial proportion of mothers fed their infants expressed breast milk exclusively at three months postpartum and beyond. In this study, we explore the predictors of mode of breastfeeding and examine its association with duration of breastfeeding in the same cohort.

## 2. Materials and Methods

### 2.1. Study Design and Population

Participants in the Growing Up in Singapore Toward healthy Outcomes (GUSTO) birth cohort study were recruited between 2009 and 2010 from KK Women’s and Children’s Hospital (KKH) and National University Hospital (NUH) in Singapore. Details of the study are described elsewhere [16]. Briefly, a total of 1247 women aged 18–46 years and of homogeneous (both parents) Chinese, Malay or Indian ethnicity were recruited during their first trimester pregnancy. The study was approved by the National Healthcare Group Domain Specific Review Board (NHG DSRB) and the Sing Health Centralised Institutional Review Board (CIRB). Written informed consent was provided by all participants.

### 2.2. Data Collection

Information on participants’ ethnic backgrounds, highest education attainment and working status were obtained from recruited women through interviewer-administered questionnaires conducted during the first trimester of pregnancy (<14 weeks). Maternal pre-pregnancy BMI was derived from self-reported pre-pregnancy weight and standing height measured at 26–28 weeks of pregnancy using a stadiometer (SECA model 213, SECA Corp., Hamburg, Germany). Details of delivery such as delivery mode, parity, gestational age, infant sex and child’s birth weight were extracted from medical records. Infants were classified into birth weight percentiles using the procedure of Mikolajczyk et al. (2011) [17]. 

### 2.3. Assessment of Breastfeeding Variables 

Women were asked about their breastfeeding intentions at 26–28 weeks’ gestation. At 3 weeks postpartum, mothers’ breastfeeding practices were captured using interviewer-administered questionnaires; these data included whether the mother experienced pain when breastfeeding (yes, no), the mother’s perception of her milk yield (more than enough, just enough, not enough), the baby’s breastfeeding schedule (on demand, regularly 2–3 hourly, regularly ≥4 hourly or irregularly) and whether the mother breastfed her baby at night (yes, no). The type of infant feeding (exclusive breastfeeding, predominant breastfeeding, partial breastfeeding vs. formula only) and data pertaining to the age at which breastfeeding ceased were captured at every postnatal visit at Week 3, Month 3 and at every 3-month interval until Month 12. The age at introduction of solid foods was ascertained at the 9-month visit. At 3 months, mothers were asked about their mode of breastfeeding (direct breastfeeding vs. expressed breast milk vs. mixed feeding, where mixed feeding refers to an infant who was both fed directly at the breast and received expressed breast milk) and the main reason for breast milk expression, if the mother mentioned that her infant was fed expressed breast milk to any extent. 

As detailed previously [15], infants who were *fully breastfed* were those who received breast milk (including expressed breast milk or milk from a wet nurse), certain liquids (water and water-based drinks, fruit juices), or syrups and drops consisting of vitamins, minerals and medicines and oral rehydration salts (ORS) solution. *Any breastfeeding* refers to an infant receiving breast milk (including expressed breast milk or milk from a wet nurse), with or without non-human milk and/or solids. Only one infant in our study, who was partially breastfed, received breast milk from a wet nurse. The mode of breastfeeding refers to the method in which an infant was fed breast milk, regardless of whether he/she consumed non-human milk and/or solids. *Direct breastfeeding* refers to an infant who was fed directly at the breast. *Expressed breast milk (EBM)* only refers to an infant who received breast milk expressed from the breast (either manually or via a pump), by bottle, cup or spoon. *Mixed feeding* refers to an infant who was fed directly at the breast and received expressed breast milk. 

### 2.4. Statistical Analyses

**Figure 1:**
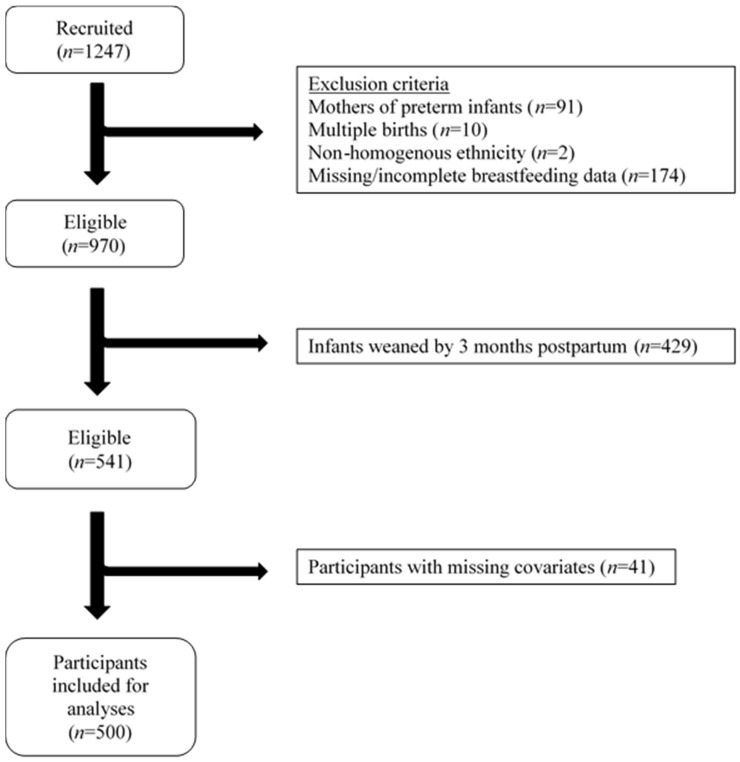
Flowchart of participants included for analysis in the GUSTO (Growing Up in Singapore Towards healthy Outcomes) study, Singapore.

Of those recruited, women who delivered preterm (<37 weeks’ gestation), had multiple pregnancies, or had incomplete information on breastfeeding mode and duration were excluded from analyses. As 3 months postpartum was the earliest time point when mode of breastfeeding was recorded, infants who were weaned by 3 months postpartum were also excluded from analyses (Figure 1). Hence, 541 participants remained eligible for the present analysis. 

Characteristics and breastfeeding practices of participants are described as proportions or means, with comparisons among the three modes of breastfeeding based on chi-square tests or ANOVA, respectively. 

Multivariable ordinal logistic regression analyses were used to identify predictors of breast milk expression, where the levels of breast milk expression in ascending order were defined as: *direct breastfeeding < mixed feeding* < *expressed breast milk only*. The test of parallel lines (proportional odds) was not significant (*p* = 0.168), indicating that model assumptions were not violated. The inclusion of factors in the multivariable models were based on previous studies [3,18] and included: ethnicity (Chinese, Malay, and Indian), maternal age (<30, 30–35, and ≥35 years old), maternal education (secondary education or below, technical college/pre-university, university), pre-pregnancy BMI (Asian BMI cutoffs of <18.5, 18.5–23, 23–27.5, and ≥27.5 kg/m^2^), parity (primipara and multipara), child’s sex, gestational age in completed weeks (continuous), birth weight category (small for gestational age (SGA), appropriate for gestational age (AGA), large for gestational age (LGA)), mode of delivery (vaginal and caesarean) and working during first trimester pregnancy (no, yes). 

Multivariable Cox regression analyses were performed to examine whether the mode of breastfeeding was associated with the duration of any and of full breastfeeding. Variables included in these models were: ethnicity (Chinese, Malay, and Indian), maternal age (continuous), maternal education (secondary education or below, technical college/pre-university, and university), pre-pregnancy BMI (<18.5, 18.5–23, 23–27.5, ≥27.5 kg/m^2^), parity (primipara and multipara), child’s sex, birth weight category (SGA, AGA, and LGA), and working during first trimester pregnancy (no and yes). We also conducted additional multivariable Cox regression analyses that further adjusted for the reasons for breast milk expression (Supplementary Table S1), and tested for effect modification by the reasons for breast milk expression by including a multiplicative interaction term into the multivariable model. 

As the proportion of covariates with missing data was low, ranging from 0.7% to 6.7%, we excluded participants with missing covariates from the statistical analyses. 

All statistical analyses were performed using SPSS version 20.0 (IBM Corp., Armonk, NY, USA).

## 3. Results

Of the participating mothers still breastfeeding three months post-delivery, most were either breastfeeding their infants directly at the breast (43.3%) or practicing mixed feeding (40.3%); a smaller proportion of mothers fed their infants EBM only (16.5%). Chinese mothers, mothers with university education, first-time mothers and mothers who worked during their pregnancy tended to feed their infants EBM only at three months postpartum instead of direct breastfeeding (Table 1). With regard to early breastfeeding behaviours, a higher proportion of mothers who perceived that their milk production was insufficient to meet the demands of her child, those who fed their infants regularly at 2–3 hourly intervals, and those who did not breastfeed at night three weeks postpartum, fed their infants EBM exclusively. Duration of any and of full breastfeeding were shortest for the group of mothers who fed their infants expressed breast milk only.

Table 2 shows the significant predictors of breast milk expression. Adjusted ordinal logistic regression analysis revealed that several maternal factors were independently associated with increasing level of breast milk expression at three months postpartum. These included Chinese (vs. Indian) ethnicity (adjusted odds ratio (OR), 95% CI; 3.41, 1.97–5.91), tertiary education (vs. secondary education or lower) (2.22, 1.22–4.04), primiparity (1.54, 1.04–2.26) and working during the first trimester of pregnancy (2.53, 1.60–4.02). 

Table 3 shows the associations of breastfeeding mode with the time to cessation of any and of full breastfeeding. Among all mothers still breastfeeding (to any degree) at three months post-delivery, those who fed their infants EBM only (vs. those who breastfed their infants directly) had a higher hazard of earlier termination of any breastfeeding (adjusted hazard ratio (HR), 95% CI; 2.20, 1.61–3.02) (Table 3). A similar association was seen among mothers fully breastfeeding at three months (2.39, 1.05–5.41). Total expression of breast milk, however, was not associated with earlier termination of full breastfeeding among mothers who were already fully breastfeeding at three months post-delivery (0.93, 0.48–1.79). Mothers who practiced mixed feeding were not at higher risk of earlier termination of any or of full breastfeeding vs. those who fed their infants directly at the breast.

The associations of breastfeeding mode with duration of any breastfeeding remained similar when we further adjusted for the reasons for breast milk expression among all mothers who were breastfeeding at three months postpartum (Supplementary Table S1), and albeit attenuated, the association was also similar among mothers who were fully breastfeeding. The reasons for breast milk expression did not appear to significantly modify the association between mode of breast milk feeding and duration of any breastfeeding among all breastfeeding mothers (*p*-interaction = 0.16) and among those who were fully breastfeeding (*p*-interaction = 0.47). Similarly, effect modification by the reasons for breast milk expression was not significant for the association between mode of breast milk feeding and duration of full breastfeeding (*p*-interaction = 0.38).

## 4. Discussion

In this prospective cohort study, we found that expression of breast milk is common among multi-ethnic Asian women. Women who were of Chinese descent, with higher educational attainment, first-time mothers and those who worked during early pregnancy were more likely to feed their infants EBM. Importantly, our results indicate that women who fed their infants EBM only at 3 months postpartum had a higher risk of early weaning compared to those who fed their infants directly at the breast. Women who practiced mixed feeding were not at higher risk of early weaning.

At three months postpartum, 56.6% of mothers were feeding their infants EBM to some extent. Several factors were found to be independently associated with more breast milk expression in our population. These include employment during early pregnancy and being a first-time mother, results consistent with findings of previous studies [3,4,12,18]. 

We found that maternal education attainment was associated with higher levels of breast milk expression, which differs to a recent study from Hong Kong [12]. However, this difference in results is attributed to differences in outcomes examined (increasing levels of breast milk expression vs. exclusive breast milk expression), as well as the statistical model used: predictors of increasing levels of breast milk expression (ordinal logistic regression) vs. predictors of exclusive breast milk expression (logistic regression); maternal education attainment was no longer associated with breast milk expression in our study when we employed the same statistical model and examined the same outcome as Bai et al. (2016) [12]. It is unclear why mothers of higher education attainment tend to express breast milk. As higher education attainment is often associated with higher income, affordability of breast pumps is a possible reason for the association of education with breast milk expression. However, further adjustment of household income in the model did not attenuate the association between higher education and breast milk expression (results not shown). More work is required to understand why mothers with higher education attainment tend to express breast milk as compared to those with lower education attainment in this analytical population. It is worth acknowledging that a large proportion of mothers with lower education attainment have been excluded from this present study as they had terminated breastfeeding by three months postpartum [15]. In this respect, it appears that women with a higher education may be more motivated to express breast milk when there are hurdles to direct breastfeeding whereas women with a lower education who face the same hurdles may not breastfeed at all (i.e., not be in the current analytical population).

Another determinant of breast milk expression in our study was ethnicity; mothers of Chinese ethnicity were more likely to express breast milk at three months postpartum than Malay or Indian mothers. To our knowledge, this is the first study of breast milk expression in women from different Asian ethnicities. We have previously speculated on why Chinese mothers tend to express breast milk more than mothers of other ethnicities [15] and suggested that cultural factors, such as embarrassment to breastfeed in public [19], may be the main reason. Further qualitative studies need to be conducted to explore and determine the reasons behind the association.

Girls were more likely to be fed directly at the breast when compared to boys. The reasons for this difference are unclear, as no significant differences were observed in parity, ethnicity or working status among mothers who had a boy vs. a girl. Additionally, in both unadjusted and adjusted ordinal logistic regression models, infant sex was not predictive of increasing levels of breast milk expression. Studies in other populations would help sort out whether this is a chance finding or reflects a true maternal preference. 

We found that mode of breast milk feeding was related to breastfeeding duration, with mothers who exclusively fed their infants expressed breast milk at three months postpartum having a higher incidence of early weaning compared to those who fed their infants directly at the breast. This observation is consistent with those reported previously in Shanghai, Hong Kong, and the U.S. [4,12,20], despite differences in time at which expression of breast milk was ascertained (ranging from one to six months postpartum). Even among our study mothers who were fully breastfeeding, those who expressed breast milk exclusively at three months postpartum had a higher hazard of early weaning than those who breastfed directly. The association may be driven by mothers who have difficulties feeding their child directly at the breast (e.g., mothers with inverted/flat nipples), since previous studies have shown that these mothers breastfed for shorter duration [21,22]. However, mothers in our study who expressed breast milk exclusively did so for many reasons, including elective reasons (e.g., wanting to know the volume of milk produced), and the risk of early weaning was independent of the reasons for breast milk expression. In contrast, mothers who practiced mixed feeding were not at a higher risk of early weaning when compared to those who were fed exclusively at the breast. Several studies have examined this association, with inconsistent results [4,12,13,14,18,23]. Differences that may explain some of the inconsistencies in results between studies include the definition of mixed feeding [13,14,18] and the time of collecting data on breast milk expression, which ranged from 24–48 h [13] to six weeks after delivery [4], to any breast milk expression in the first six months of infant’s life [14]. A number of factors may explain why mixed feeding was not associated with higher risk of early weaning vs. direct breastfeeding in our study. Mixed feeding allows mothers who return to work to continue providing breast milk for their infants. The expression of breast milk also facilitates maternal independence by releasing the mother from the constant demands of her infant [24], which in turn may have a positive impact on breastfeeding duration. 

Strengths of our study include its prospective design, inclusion of three Asian ethnicities, and comprehensive data collected on potential confounders. Breastfeeding status was documented three-monthly, reducing recall bias. Limitations to our study include the lack of information on employment status at three months postpartum. However, we accounted for the effect of working status in the models by adjusting for the women’s working status during pregnancy, which correlated strongly with their working status at six months postpartum (*r* = 0.96). Second, breastfeeding data were missing for 174 participants; mothers with missing data were younger and had lower educational attainment, and their infants were of lower birth weight. Our results may therefore not be generalizable to the entire local population. Third, we did not collect data on mode of breastfeeding at time points earlier than three months post-delivery and hence are unable to confirm whether the association of mode of breastfeeding with breastfeeding duration was also present at earlier time points. 

## 5. Conclusions

In conclusion, over 50% of breastfeeding mothers in our multi-ethnic Asian cohort fed their infants expressed breast milk to some extent at three months postpartum, and, of these, 16% fed expressed breast milk exclusively. Mothers who expressed breast milk only for their infants, but not those who practiced mixed feeding, were at greater risk of terminating breastfeeding earlier than those who breastfed directly. More education and support are required for women feeding their infants expressed breast milk only, especially those with other risk factors for early weaning. Given its association with breastfeeding duration, and the scarcity of research on mode of breastfeeding, future work should examine associations between breastfeeding mode and child health outcomes, including cognitive function, immunity and growth—an area of work which we plan to explore in the GUSTO cohort. 

## Figures and Tables

**Table 1:** Characteristics and breastfeeding practices reported at three weeks among mothers practicing different breastfeeding modes at three months postpartum (*n* = 500) ^1^.

	All Participants (*n* = 500)	Direct Breastfeeding (*n* = 217, 43.4%)	Mixed Feeding (Direct and Expressed) (*n* = 200, 40.0%)	Expressed Breast Milk Only (*n* = 83, 16.6%)	*p*
Maternal Age (years), Mean ± SD	31.7 ± 4.6	31.9 ± 5.0	31.6 ± 4.3	31.6 ± 4.2	0.657
Pre-pregnancy BMI (kg/m^2^)					0.432
<18.5	62	21 (33.9)	30 (48.4)	11 (17.7)	
18.5–<23	273	114 (41.8)	113 (41.4)	46 (16.8)	
23–<27.5	122	59 (48.4)	43 (35.2)	20 (16.4)	
≥27.5	43	23 (53.5)	14 (32.6)	6 (14.0)	
Ethnicity					<0.001
Chinese	322	102 (31.7)	150 (46.6)	70 (21.7)	
Malay	90	57 (63.3)	28 (31.1)	5 (5.6)	
Indian	88	58 (65.9)	22 (25.0)	8 (9.1)	
Maternal Education					<0.001
No education/Primary/Secondary	82	57 (69.5)	18 (22.0)	7 (8.5)	
Technical College/Pre-university	152	70 (46.1)	60 (39.5)	22 (14.5)	
University	266	90 (33.8)	122 (45.9)	54 (20.3)	
Parity					<0.001
Primipara	230	76 (33.0)	100 (43.5)	54 (23.5)	
Multipara	270	141 (52.2)	100 (37.0)	29 (10.7)	
Gestational Age (weeks), Mean ± SD	39.0 ± 1.0	39.0 ± 1.0	38.9 ± 1.0	39.1 ± 0.9	0.269
Child’s Birth Weight (g), Mean ± SD	3167.3 ± 384.3	3135.5 ± 361.9	3194.2 ± 401.6	3185.9 ± 397.0	0.265
Child’s Birth Weight Percentile					0.605
SGA (<10%)	55	28 (50.9)	17 (30.9)	10 (18.2)	
AGA (10–90%)	365	158 (43.3)	148 (40.5)	59 (16.2)	
LGA (>90%)	80	31 (38.8)	35 (43.8)	14 (17.5)	
Child’s sex					0.022
Male	264	101 (38.3)	120 (45.5)	43 (16.3)	
Female	236	116 (49.2)	80 (33.9)	40 (16.9)	
Delivery Mode					0.966
Vaginal	355	155 (43.7)	142 (40.0)	58 (16.3)	
Caesarean Section	145	62 (42.8)	58 (40.0)	25 (17.2)	
Working during 1st Trimester Pregnancy					<0.001
No	130	89 (68.5)	29 (22.3)	12 (9.2)	
Yes	370	128 (34.6)	171 (46.2)	71 (19.2)	
Planned to Breastfeed					0.863
No	6	2 (33.3)	3 (50.0)	1 (16.7)	
Yes	494	215 (43.5)	197 (39.9)	82 (16.6)	
Experienced Pain When Breastfeeding ^3^					0.537
No	184	84 (45.7)	74 (40.2)	26 (14.1)	
Yes	296	126 (42.6)	117 (39.5)	53 (17.9)	
Mother‘s Perceived Milk Yield ^2,3^					<0.001
More than Enough	132	49 (37.1)	72 (54.5)	11 (8.3)	
Just Enough	194	92 (47.4)	78 (40.2)	24 (12.4)	
Not Enough	153	68 (44.4)	43 (28.1)	42 (27.5)	
Breastfeeding Schedule ^2,3^					0.001
On Demand	261	120 (46.0)	112 (42.9)	29 (11.1)	
Regularly 2–3 Hourly	164	59 (36.0)	68 (41.5)	37 (22.6)	
Regularly 4 Hourly, or Irregularly	52	30 (57.7)	12 (23.1)	10 (19.2)	
Breastfed at Night ^2,3^					<0.001
No	73	17 (23.3)	21 (28.8)	35 (47.9)	
Yes	409	193 (47.2)	172 (42.1)	44 (10.8)	
Duration of Any Breastfeeding, Mean ± SD	9.2 ± 4.4	9.5 ± 4.7	9.7 ± 4.2	7.1 ± 3.6	<0.001
Duration of Full Breastfeeding, Mean ± SD	2.3 ± 2.1	2.3 ± 2.0	2.5 ± 2.2	1.6 ± 1.6	0.001

^1^ Data presented are *n* (%) unless otherwise stated. ^2^ Number of participants with missing data: Mother’s perceived milk yield (*n* = 21); Breastfeeding schedule (*n* = 23); Breastfed at night (*n* = 18). ^3^ At three weeks post-delivery. AGA: appropriate for gestational age; LGA: large for gestational age; SGA: small for gestational age.

**Table 2:** Factors independently associated with increasing levels of breast milk expression at three months post-delivery (*n* = 500).

	*n*	Unadjusted OR (95% CI)	Adjusted ^1^ OR (95% CI)
Ethnicity			
Chinese	322	3.97 (2.44, 6.43)	3.41 (1.97, 5.91)
Malay	90	1.05 (0.57, 1.92)	1.08 (0.54, 2.19)
Indian	88	Reference	Reference
Maternal Education			
No Education/Primary/Secondary	82	Reference	Reference
Technical College/Pre-university	152	2.58 (1.49, 4.49)	1.39 (0.76, 2.54)
University	266	4.17 (2.48, 6.99)	2.22 (1.22, 4.04)
Parity			
Primipara	230	2.30 (1.64, 3.23)	1.54 (1.04, 2.26)
Multipara	270	Reference	Reference
Working during 1st Trimester Pregnancy			
No	130	Reference	Reference
Yes	370	3.79 (2.50, 5.74)	2.53 (1.60, 4.02)

^1^ Ordinal logistic regression adjusted for maternal age (<30, 30–35, ≥35 years old), ethnicity (Chinese, Malay, Indian), maternal education (no education/primary/secondary, technical college/pre-university, university), pre-pregnancy BMI (<18.5, 18.5–23, 23–27.5, ≥27.5 kg/m^2^), parity (primipara, multipara), child’s sex (male, female), gestational age (continuous), birth weight category (SGA, AGA, LGA), mode of delivery (vaginal, caesarean section) and working during 1st trimester pregnancy (no, yes). (Test to reject assumption of parallel lines (proportional odds): *p* = 0.168).

**Table 3:** Associations of the mode of breastfeeding at 3 months post-delivery on the risk of terminating any and full breastfeeding.

		Terminating any Breastfeeding		Terminating Full Breastfeeding	
		Unadjusted	Adjusted ^1^	Unadjusted	Adjusted ^1^
	*n*	HR (95% CI)	HR (95% CI)	HR (95% CI)	HR (95% CI)
All mothers Breastfeeding at 3 Months Post-delivery					
Direct Breastfeeding	217	Reference	Reference	-	-
Mixed Feeding	200	1.15 (0.91, 1.46)	1.17 (0.90, 1.52)	-	-
Expressed Breast Milk Only	83	2.17 (1.62, 2.89)	2.20 (1.61, 3.02)	-	-
Mothers Fully Breastfeeding at 3 Months Post-delivery					
Direct Breastfeeding	72	Reference	Reference	Reference	Reference
Mixed Feeding	76	1.27 (0.82, 1.97)	1.28 (0.75, 2.17)	0.92 (0.66, 1.28)	0.90 (0.62, 1.30)
Expressed Breast Milk Only	11	1.77 (0.82, 3.81)	2.39 (1.05, 5.41)	0.94 (0.50, 1.78)	0.93 (0.48, 1.79)

^1^ Full model adjusted for maternal age (continuous), ethnicity (Chinese, Malay, Indian), maternal education (secondary education or below, technical college/pre-university, university), parity (primipara, multipara), pre-pregnancy BMI (continuous), birth weight category (SGA, AGA, LGA), child’s sex (male, female) and working during 1st trimester pregnancy (no, yes).

## Supplementary Materials

The following are available online at www.mdpi.com/2072-6643/9/6/547/s1, Table S1: Associations of the mode of breastfeeding at 3 months post-delivery on the risk of terminating any and full breastfeeding, with further adjustment for the reasons for breast milk expression.
