# Supplementary material for: Direct vs. Expressed Breast Milk Feeding: Relation to Duration of Breastfeeding
Source: Nutrients. 2017 May 27;9(6):547. doi: 10.3390/nu9060547 (PMC5490526; doi:10.3390/nu9060547)
Supplement: Supplementary file 1 [file nutrients-09-00547-s001.pdf]

Supplementary table S1. Associations of the mode of breastfeeding at 3 months post-delivery on the risk of terminating any and full breastfeeding, with further adjustment for the reasons for breast milk expression.

		Terminating any breastfeeding	Terminating full breastfeeding
		Adjusted[1]	Adjusted[1]
	n	HR (95% CI)	HR (95% CI)
All mothers breastfeeding at 3 months post-delivery			
Direct breastfeeding	217	Reference	
Mixed feeding	198	1.22 (0.74, 2.03)	
Expressed breast milk only	82	1.85 (1.05, 3.28)	
Mothers fully breastfeeding at 3 months post-delivery			
Direct breastfeeding	72	Reference	Reference
Mixed feeding	76	1.00 (0.43, 2.28)	1.18 (0.65, 2.16)
Expressed breast milk only	11	1.66 (0.52, 5.34)	1.28 (0.52, 3.18)

[1]Full model included adjustments for maternal age (continuous), ethnicity (Chinese, Malay, Indian), maternal education (secondary education or below, technical college/pre-university, university), parity (primipara, multipara), pre-pregnancy BMI (continuous), birth weight category (SGA, AGA, LGA), child's sex (male, female), working during 1st trimester pregnancy (no, yes) and reasons for breast milk expression (has excess breast milk/ to store for future use or for work, all other reasons).
